# Oral nystatin prophylaxis to prevent systemic fungal infection in very low birth weight preterm infants: a randomized controlled trial

**DOI:** 10.1186/s12887-020-02074-0

**Published:** 2020-04-17

**Authors:** Lily Rundjan, Retno Wahyuningsih, Chrissela Anindita Oeswadi, Miske Marsogi, Ayu Purnamasari

**Affiliations:** 1 Division of Neonatology, Department of Pediatrics, Faculty of Medicine, Universitas Indonesia - Cipto Mangunkusumo Hospital, Jalan Diponegoro 71, DKI Jakarta, 10430 Indonesia; 2 Division of Mycology, Department of Parasitology, Faculty of Medicine, Universitas Indonesia - Cipto Mangunkusumo Hospital, DKI Jakarta, Indonesia

**Keywords:** Nystatin, Fungal colonization, Systemic fungal infection

## Abstract

**Background:**

Systemic fungal infection (SFI) is one of leading causes of morbidity and mortality in very low birth weight (VLBW) preterm infants. Because early diagnosis of SFI is challenging due to nonspecific manifestations, prophylaxis becomes crucial. This study aimed to assess effectiveness of oral nystatin as an antifungal prophylaxis to prevent SFI in VLBW preterm infants.

**Methods:**

A prospective, open-labelled, randomized controlled trial was performed in a neonatal intensive care unit (NICU) of an academic hospital in Indonesia. Infants with a gestational age ≤ 32 weeks and/or birth weight of ≤ 1500 g with risk factors for fungal infection were assessed for eligibility and randomized to either an intervention group (nystatin) or control group. The intervention group received 1 ml of oral nystatin three times a day, and the control group received a dose of 1 ml of sterile water three times a day. The incidence of fungal colonization and SFI were observed and evaluated during the six-week study period. Overall mortality rates and nystatin-related adverse drug reactions during the study period were also documented.

**Results:**

A total of 95 patients were enrolled. The incidence of fungal colonization was lower among infants in nystatin group compared to those in control group (29.8 and 56.3%, respectively; relative risk 0.559; 95% confidence interval 0.357–0.899; p-value = 0.009). There were five cases of SFI, all of which were found in the control group (p-value = 0.056). There was no difference in overall mortality between the two groups. No adverse drug reactions were noted during the study period.

**Conclusions:**

Nystatin is effective and safe as an antifungal prophylactic medication in reducing colonization rates in the study population. Whilst the use of nystatin showed a potential protective effect against SFI among VLBW preterm infants, there was no statistical significant difference in SFI rates between groups.

**Trial registration:**

NCT03390374. Registered 4 January 2018 - Retrospectively registered.

## Background

Systemic fungal infection (SFI) is a form of late onset neonatal sepsis (LOS) that accounts for 12% of all LOS among very low birth weight (VLBW or < 1500 g birth weight) [1]. The incidence of SFI varies between 1 and 10% in VLBW and 2–26% in extremely low birth weight (ELBW or < 1000 g birth weight) infants [1–7]. In comparison, the incidence of SFI in our hospital was 26% in VLBW infants between years 2005 and 2008 [8].

Systemic fungal infection is a significant problem in neonatal care due to mortality rates that are comparable with Gram-negative infections, both being 3–4 fold higher than Gram-positive infections [9]. Furthermore, SFI is associated with adverse long-term neurodevelopmental outcomes among survivors [2, 3, 9–11]. Prevention of SFI has become crucial because rapid diagnosis of SFI remains challenging. This is due to its nonspecific clinical presentation and low yields of fungal isolation in culture, all of which may result in a delay in instituting treatment [7, 12].

The most common causative agent of SFI is *Candida* spp. [1, 9, 13], with *C. albicans* being the most common vertically transmitted species and *C. parapsilosis* mostly responsible for horizontal transmission. Other fungal species can occasionally be found in particular condition, such as *Malassezia furfur*, which has been associated with the use of parenteral lipid nutrition, and *Aspergillus* spp., which is found predominantly in infants with local skin trauma [14].

*Candida* spp. is a commensal organism with ability to adhere to human epithelium, particularly in gastrointestinal (GI) tract, leading to colonization [11]. About 88% of fungal colonizations are detected on the third day after birth, mostly in anus (88.8%), oral cavity (66.6%), and umbilicus (55%) [15]. This GI colonization, especially heavy colonization, may become a primary source of translocation through epithelial barriers and systemic dissemination in high-risk VLBW preterm infants due to compromised mucosal integrity or host defences [11–18]. Additional factors that increase the risk factors for fungal colonization and SFI are the presence of central catheters or endotracheal tubes, prolonged use of parenteral nutrition, delayed enteral feeding, and also the use of certain medications such as broad spectrum antibiotics, corticosteroids, theophylline, and histamine type 2 receptor blocker [1, 2, 6, 16]. Theophylline is known to inhibit neutrophil mediated damage to *Candida albicans* pseudohyphae [17, 18]. Because Histamine 2 receptor blockers elevate gastric pH, their use increases the risk of fungal overgrowth in gastrointestinal tract [19].

Prevention of *Candida* colonization in the GI tract using systemic or oral non-absorbable antifungal prophylaxis has been considered to be effective in decreasing SFI among high-risk infants [11]. A meta-analysis of fluconazole prophylaxis demonstrated a marked reduction in the risk of severe fungal infection in VLBW preterm infants [20]. Nevertheless, there have been several reports of emergence of fungal resistance and hepatotoxicity associated with the use of fluconazole [5, 13, 21–24]. A comparable prophylactic effect has also been seen with use of oral nystatin. Oral nystatin is not systemically absorbed, allowing sufficient contact with colonizing fungal agents in the GI tract which is the main portal of entry [25, 26]. Although it is non-toxic, easy to use, and less expensive [20, 21, 25–30], the validity and applicability of the latest meta-analysis of nystatin prophylaxis [31] has been limited due to insufficient data and methodological problems (e.g. concealment, allocation, and blinding problems) [31, 32]. To date, 14 fluconazole trials were registered compared to five nystatin trials.

In our unit, there has been a documented emergence of *Non-albicans Candida* (NAC) species, that is, any *Candida* spp. infection except *Candida albicans* [33], with possibility of reduced susceptibility to fluconazole in our patient population. Additionally, we have a limited budget for the provision of medications in our unit, and ongoing difficulties in maintaining intravenous access for systemic antifungal agents. For this reason, we aimed to determine the effectiveness of oral nystatin as an alternative agent for antifungal prophylaxis among VLBW preterm infants in our unit.

## Methods

### Study design

A prospective, open-labelled, randomized controlled trial was conducted to evaluate the effectiveness of nystatin prophylaxis among preterm and/or VLBW infants. This study was reviewed and approved by the Committee of the Medical Research Ethics of the Faculty of Medicine Universitas Indonesia. This trial has been retrospectively registered in clinicaltrials.gov with trial registration number NCT03390374. We adhered to the CONSORT guideline in reporting this trial’s results.

### Study setting and study population

This trial was conducted at Cipto Mangunkusumo Hospital, an academic National Hospital in Indonesia, which provides tertiary neonatal care. From 2010 to 2012, eligibility was assessed among all inborn infants admitted to our neonatal intensive care unit within the first 72 h of life who had a gestational age of ≤ 32 weeks and/or birth weight of ≤ 1500 g. During the study period infants < 28 weeks or < 1000 g had to be excluded due to the very poor survival of these infants before the nystatin could be administered. Infants included also had one or more SFI risk factors present (antibiotic therapy, intravenous access, endotracheal tube, orogastric tube, urinary catheter, corticosteroid therapy, parenteral nutrition, and theophylline therapy). All infants suspected of having necrotizing enterocolitis (NEC) within 72 h after birth, cyanotic congenital heart disease, chromosomal defects, or other critical conditions with poor prognosis were excluded. Written informed consents were obtained prior to recruitment.

### Study intervention

Enrolled infants were randomly assigned into the nystatin or control groups. Infants in the nystatin group received oral nystatin (Mycostatin® oral suspension 100.000 U/mL, manufactured by Taisho Pharmaceuticals Indonesia) with a dosage of 1 mL (0.5 mL was coated in oral cavity and another 0.5 mL was given through orogastric tube) three times a day for the six weeks of the study period or until no risk factors of SFI were noted. If any sign of GI bleeding was noted, orogastric nystatin administration was suspended and only oral coating with nystatin was continued. Nystatin was discontinued entirely if there was any concern of NEC or shock. The control group received 1 mL of sterile water three times a day as a coating in oral cavity as according to our protocol of oral hygiene care.

Oropharyngeal and perianal fungal swabs were collected weekly for the purpose of direct microscopic examination and culture to identify GI colonization. These specimens were analyzed in Department of Parasitology, Faculty of Medicine, Universitas Indonesia. In order to diagnose SFI, culture of blood, cerebrospinal fluid (CSF), endotracheal tube aspirate, and urine were done if the patient developed clinical signs of fungal sepsis, fungal meningitis, fungal pneumonia, or fungal urinary tract infections. Once the diagnosis of SFI was established, systemic therapy for fungal infection was commenced using intravenous Amphotericin B. Nystatin was discontinued if the patient no longer had any risk factor for SFI, was discharged from the hospital, or died.

### Randomization

Enrollment and randomization were carried out by co-investigators. Once the parents were consented for the study, co-investigators randomly assigned the infants by using simple randomization method at 1:1 ratio. A sealed opaque envelope was removed from a closed container and opened to determine the group allocation. The allocated treatment regimen was then applied to the infants. Study investigators and attending care teams were not intentionally blinded to treatment allocation due to inability to provide a nystatin placebo. To reduce risk of bias due to unblinding, the laboratory personnel who analyzed outcome were not informed of treatment allocation and results of colonization were only known by investigators who were not involved in clinical decision making.

### Study outcomes

The primary outcome of this study was the incidence of fungal colonization. Weekly oropharyngeal and perianal fungal swabs for direct microscopic examination and culture were assessed to determine fungal colonization. The result was considered positive if fungal elements were isolated on either oropharyngeal or perianal specimens. Colonization was then graded into light (< 10 colonies), moderate (10–99 colonies), or heavy (> 99 colonies) [34, 35]. Time of onset of colonization, sites involved, and fungal organism isolated at the colonization site were also documented. Culture results were evaluated if the infants’ condition worsened to ascertain whether they met the criteria of SFI. Proven SFI was defined as a positive fungal culture from blood, CSF, endotracheal tube aspirate, deep tissue, or urine (> 10,000 or more colony-forming unit/mL from sterile bladder catheterization or suprapubic aspiration).

As secondary outcomes, SFI, overall mortality rates and nystatin-related adverse drug reactions (such as vomiting, diarrhea, and allergic reaction) during the study period were documented. Fungal-related mortality was defined as mortality that occurred within 72 h of positive fungal blood culture or positive evidence of disseminated candidiasis on autopsy.

### Sample size and statistical analysis

It was estimated that 35 subjects would be required in each group to detect an absolute 5% decrease in the primary outcome among the two groups, with two-tailed α of 0.05 and power of 80%. Data analysis was conducted using an intention-to-treat approach. Baseline data on characteristics and risk factors were reported as descriptive statistics including mean, median, and calculation of dispersion (standard deviation and ranges). Numerical variables were analyzed using independent-sample t-test while categorical variables using either chi-square or fisher’s exact test as appropriate. Rate analysis was done with cox regression. The results were considered statistically significant if p-value was < 0.05. Statistical analysis was performed using SPSS software 24.0 for Windows.

## Results

### Study population

**Fig. 1:**
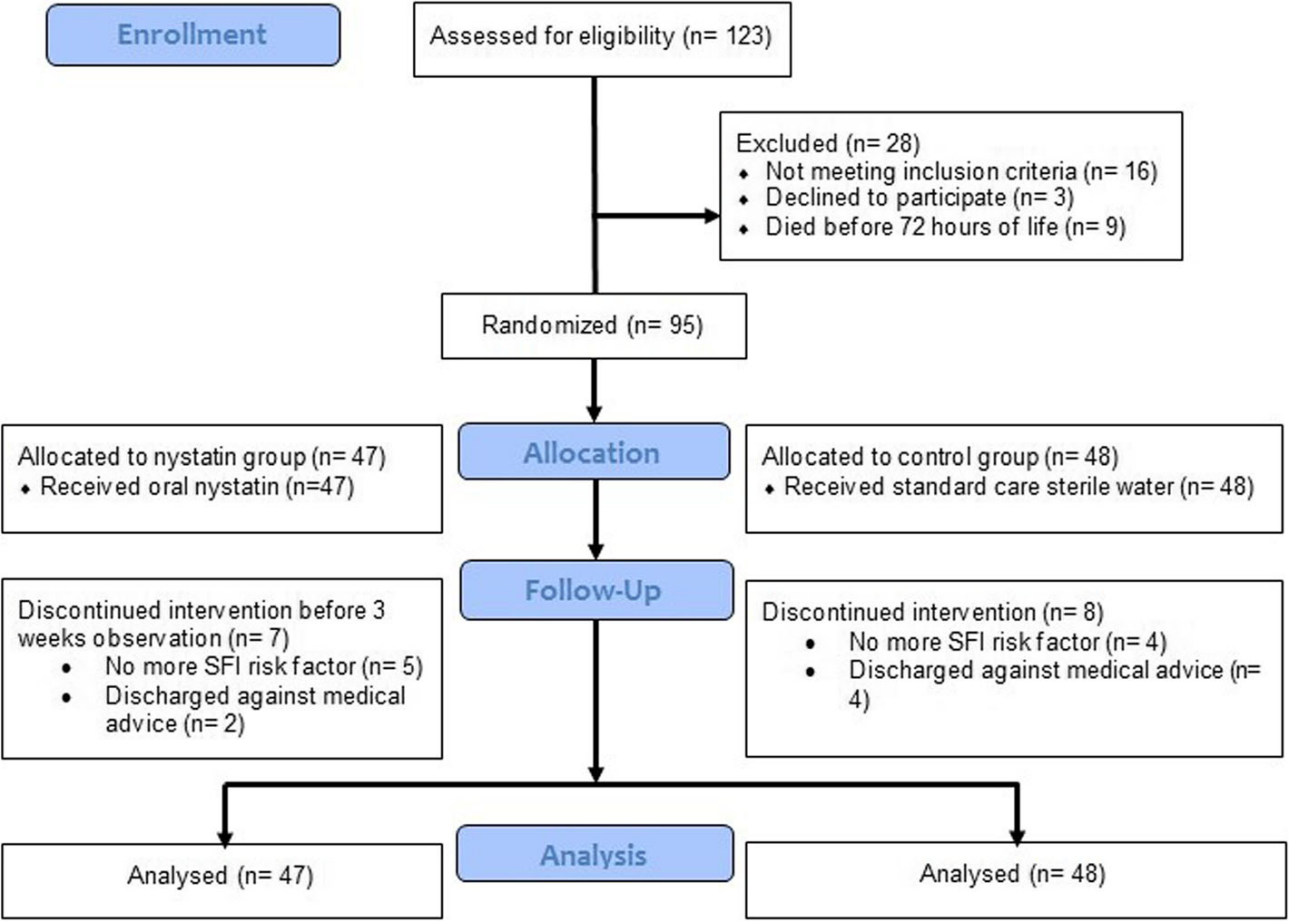
Flowchart of the participants

Between October 2010 and November 2012, a total of 123 preterm infants ≤ 32 weeks’ gestational age and/or birth weight of ≤ 1500 g were identified. Among these infants, 95 met eligibility criteria and were randomized into either nystatin group (*n* = 47) or control group (*n* = 48) (Fig. 1). Discontinuation of intervention before completion of the proposed observation period occurred in 15 infants. However, all these infants were still included in the final analysis. The trial was ended after the length of follow up had been completed. Median of follow up period was 4 weeks (range 1–6 weeks) for both groups. There was no significant difference (p-value > 0.05) in baseline neonatal characteristics and risk factors of SFI between the two groups, as shown in Tables 1 and 2, respectively.

**Table 1:** Baseline characteristics of subjects

Variables	Nystatin group (*n* = 47)	Control group (*n* = 48)
Gestational age, weeks, mean (± SD)	30.8 (±2.0)	30.5 (±2.2)
Gender (Male/Female)	24/23	32/16
Birth weight, gram, mean (± SD)	1290 (±234.6)	1318 (±259.2)
Vaginal delivery, n (%)	30 (53.2)	25 (62.5)
Apgar score at 5 min, median (range)	9 (4–10)	8 (3–10)
Rupture of membranes > 24 h, n (%)	10 (21.2)	7 (14.6)

**Table 2:** Risk factors of systemic fungal infection

Variables	Nystatin group	Control group	p-value
	(*n* = 47)	(*n* = 48)	
Duration of stay in NICU, n (%)			
Mean duration time, days, mean (± SD)	9.8 (±14.9)	13.6 (±15.5)	0.23
≤ 7 days, n (%)	8 (17.0)	8 (16.7)	0.16
> 7 days, n (%)	18 (38.3)	27 (56.3)	
Use of peripheral venous access, n (%)	47 (100)	48 (100)	1.00
Duration of peripheral venous access (days)	24 (6–42)	28 (5–62)	0.18
Use of central venous access, n (%)	38 (80.9)	37 (77.1)	0.80
Duration of central venous access (days)	19 (0–42)	19 (0–42)	0.96
Use of orogastric tube, n (%)	47 (100)	47 (97.9)	1.00
Duration of orogastric tube (days)	30 (8–42)	30 (8–42)	0.76
Use of endotracheal tube, n (%)	10 (21.3)	16 (33.3)	0.19
Duration of endotracheal tube (days)	2.6 (0–21)	4.29 (0–31)	0.26
Use of antibiotic therapy, n (%)	43 (91.5)	46 (95.8)	0.44
Duration of antibiotic therapy (days)	19 (0–42)	21 (0–42)	0.4
Use of aminophylline, n (%)	28 (59.6)	35 (72.9)	0.17
Duration (days)	11 (1–42)	13 (3–42)	0.42
Use of steroid, n (%)	3 (6.4)	3 (6.3)	0.65
Duration (days)	1 (0–12)	1 (0–3)	0.38
Use of parenteral nutrition, n (%)	45 (95.7)	47 (97.9)	0.62
Duration (days)	21 (0–41)	22 (0–42)	0.53
Duration of parenteral lipid (days)	16 (0–40)	19 (4–39)	0.15

Data are presented in median (range) or proportion

### Fungal colonization

**Fig. 2:**
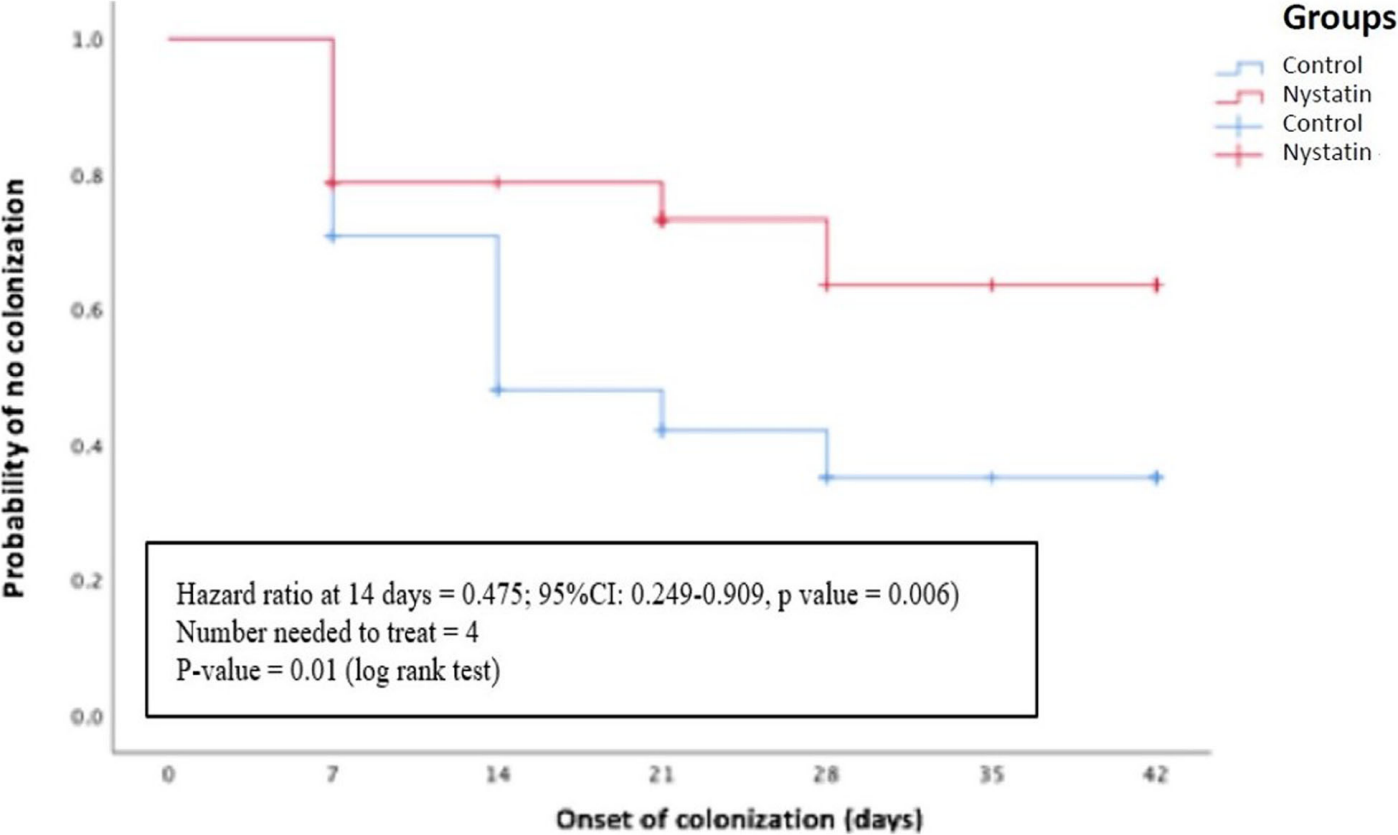
Fungal colonization over time. Hazard ratio was calculated by using time-independent cox regression

Data on fungal colonization, SFI, and mortality are presented in Table 3. The absolute fungal colonization rate was 26% lower among infants in nystatin group (29.8%) as compared to those in control group (56.3%) (RR 0.56; 95% CI 0.36–0.90, p-value = 0.009). The colonization rate was highest in the first and second week of life (Fig. 2), but the mean age of first documented colonization was similar between the two groups (11 vs 10 days, p-value > 0.05). Overtime, colonization rates during six-week observation differed significantly between the two groups (p-value = 0.01). Significantly lower rates of fungal colonization were seen after 14 days of intervention (Hazard Ratio = 0.475; 95% CI 0.249–0.909, p-value = 0.006) with a number needed to treat of four.

**Table 3:** Fungal colonization, systemic fungal infection, and mortality

Variable	Nystatin Group (*n* = 47)	Control Group (*n* = 48)	RR (95% CI)	p-value
Fungal colonization, n (%)	14 (29.8)	27 (56.3)	0.56 (0.36–0.90)	0.009
Colonization site(s)				
Single site colonization, n (%)	11 (23.4)	12 (25.0)	0.78 (0.49–1.26)	0.281
Multiple sites colonization, n (%)	3 (6.4)	15 (31.3)	0.55 (0.37–0.72)	0.001
Colonization grade				
Light colonization, n (%)	5 (10.6)	7 (14.6)	reference	
Moderate colonization, n (%)	3 (6.4)	5 (10.4)	0.93 (0.46–1.92)	1.000
Heavy colonization, n (%)	6 (12.8)	15 (31.3)	0.82 (0.47–1.42)	0.471
Systemic Fungal Infection, n (%)	0 (0)	5 (10.4)	0.09 (0.01–1.63)	0.056
Prior colonization, n/total	–	4/5		0.130
Multiple site colonization, n/total	–	2/5		0.053
Mortality				
Overall mortality, n (%)	7 (14.9)	9 (18.8)	0.86 (0.48–1.57)	0.616
Fungal-related mortality, n (%)	0	0

The number of infants with single site colonization was not significantly different between the two groups (11 vs 12 infants, p-value = 0.281). However, the incidence of multiple site colonization was significantly lower in the nystatin group (three infants; 6%) as compared to the control group (15 infants; 31%) (p-value = 0.001). There were fewer infants with heavy colonization in nystatin group compared to those in control group, although this did not reach statistical significance.

The fungal species did not differ significantly between both groups (Table 4). The most common isolated fungal species was *C. albicans* (39%). In relation to multiple species colonization, there were three infants simultaneously colonized by three different fungal species (*C. albicans*, *C. parapsilosis* and *C. kefyr* or *C. tropicalis*) and one infant colonized with 4 fungal species simultaneously (*C. albicans, C. tropicalis, C. glabrata,* and *Malassezia* spp. All these four infants were in the control group. Most of heavy colonization cases in the control group (53.3%) were caused by multiple species, whereas all cases with heavy colonization in the nystatin group were only caused by a single species (*C. albicans*).

**Table 4:** Fungal species on colonization

	Nystatin group (*n* = 14)	Control group (*n* = 27)	p-value
Single species, n (%)	11 (78.6)	17 (62.9)	0.481^c^
*Candida albicans*	7	9	
*Candida non albicans*^*a*^	2	7	
*Malassezia,* spp	2	1	
Multiple species, n (%)^b^	3 (21.4)	10 (37.0)	

^a^*C. parapsilosis/glabrata/tropicalis*

^b^*C. albicans* with *C. parapsilosis/glabrata/tropicalis/kefyr* or *Malassezia* spp.

^c^Calculated as comparison between single and multiple species

### Systemic fungal infection

During the study period there were five cases of SFI. All of them occurred in the control group but the differing incidence did not reach statistical significance as compared to the nystatin group (absolute risk reduction of 10.4%, p-value = 0.056). These cases were diagnosed by positive blood culture (three cases), positive blood and urine culture (one case), and positive intestinal tissue culture from a surgical NEC patient (one case). With respect to prior colonization, four SFI cases were preceded by colonization with the same fungal species, either light (one case) or heavy colonization (three cases), and one case was not preceded by colonization at all. There were two cases noted as having multiple site colonization prior to the development of SFI. The fungal organisms causing SFI were *C. albicans* (three cases), *C. krusei* (one case), and *C. tropicalis* (one case). These organisms were detected at a median age of 18 days (range 8–37 days).

### Overall mortality and adverse drug reaction

The overall mortality was similar between both groups (14.9% in nystatin group and 18.8% in control group, p-value = 0.616), but none of the deaths were related to fungal infection. Three major causes of overall mortality in both groups were bacterial infection, chronic lung disease, and NEC. There was no reported nystatin-related adverse drug reaction during the study period.

## Discussion

This randomized controlled trial demonstrates that oral nystatin significantly reduced the incidence of fungal colonization in our neonatal unit. Although no cases of SFI occurred in the nystatin group, the difference in SFI rates between the two groups did not reach statistical significance.

A number of risk factors have been reported to be associated with an increased risk of developing SFI. Preceding colonization, particularly in GI tract and skin, is consistently recognized as the most important predictor of SFI [23, 24, 36–38]. High rates of colonization (ranging between 22 and 87%) have been noted among preterm and VLBW infants who do not receive any antifungal prophylaxis [21, 26, 28, 30, 37, 39]. A similar result has been noted in our study, with the colonization rate in the control group being 56.3%, as compared to a 29.8% rate in the nystatin group. Density and number of colonization sites reported have a positive correlation to the risk of subsequent SFI development [24, 40], thereby increasing the risk of fungal translocation and dissemination [24, 41]. Other studies have reported that high fungal densities and multiple fungal colonization sites are associated with greater SFI risk. Kaufman, et al. demonstrated that the risk of SFI increased with each additional site colonized. The same fungal species are mostly documented in both the infection and colonization sites [37, 42, 43]. These results are similar to those reported in our study. Four of five cases in the control group were preceded by either heavy or multiple site colonization with similar fungal species. The exception was the one SFI that occurred without evidence of prior colonization.

Nystatin is a well-studied polyene antifungal that has a comparable efficacy with fluconazole for SFI prophylaxis amongst preterm and/or VLBW infants [21, 25, 44]. Previous studies have proven that nystatin can prevent the development of colonization and SFI [21, 28, 30]. A meta-analysis demonstrated that one in every 4–9 infants was prevented from developing SFI after receiving either fluconazole or nystatin prophylaxis [32]. Our study demonstrated a statistically significant reduction of fungal colonization by 26% in the nystatin group as compared to the control group. All five SFI cases in our study were in control group and no cases of SFI were found in nystatin group. Although the difference in SFI incidence between both groups was not statistically significant, this result has suggested a declining trend of SFI risk and potential preventive effect of nystatin prophylaxis against SFI. We observed that incidence of SFI in our current study (5.2%) was much lower than our previous rate in the epoch of 2005–2008 (26%) [8]. This large difference in incidence of SFI may have been influenced by a recent change in our clinical practice with the implementation of restrictive of antibiotic guidelines (narrow spectrum of antibiotics) since 2008.

The most common fungal organism cultured in this study was *C. albicans*, a finding similar to that of previous studies [26, 28, 45]. Although *C. albicans* was still the most frequently encountered fungal organism, the occurrence of NAC species (consisting of *C. parapsilosis*, *C. tropicalis*, *C. glabrata*, *C. krusei*, and *C. kefyr)* continues to increase. The shift from *C. albicans* to NAC species in our unit has been previously reported by Wahyuningsih et al. [33]. This effect may be due to long-term extensive use of fluconazole when Amphotericin B was not available during that epoch. The notable increase in the incidence of NAC may become an important determinant in selecting a prophylactic drug because some NAC species, particularly *C. glabrata* and *C. krusei*, have been reported to be natively resistant to fluconazole [24, 46, 47]. In contrast, nystatin is efficacious against all *Candida* species and no resistance has been documented with its use. *Malassezia* spp. was also found in our study. Prematurity, low birth weight, use of parenteral nutrition with lipid emulsion, use of central venous catheters, and contact with healthcare staff with contaminated hands were related to *Malassezia* spp. colonization in infants. There were some studies which have reported that this organism is an important cause of SFI and have demonstrated reduced susceptibility to fluconazole and flucytosine. To date, there are no reports of *Malassezia* spp. resistance to nystatin [48–51].

Based on the findings of the current study and previous studies, it is more difficult to reduce the risk of SFI once colonization has occurred [15, 26, 42, 46, 52]. Currently, there is no established guidelines regarding the most effective timing and duration in using nystatin prophylaxis among high-risk infants. Several previous studies suggested that colonization may start within 3–6 days of life [15, 26, 38, 53]. Therefore, to be effective in preventing fungal overgrowth and infection, oral nystatin should be commenced before the onset of colonization, particularly within the first 72 h of life [26, 52]. In contrast, the mean age of first colonization in our study was 10–11 days, longer than reported in the other previous studies. Most studies administered antifungal prophylaxis until patients were not in intensive care or no longer experiencing any risk factors for fungal infections. Although risk factors in every unit may be different, previous studies mostly agreed that antifungal prophylaxis administration for four to six weeks was safe and resulted in lower risk of colonization [5, 38, 41].

No mortality attributable to fungal infection was reported in any of our SFI cases. However, further study is still required to specifically evaluate the effect of nystatin on fungal-related mortality and long-term outcome. Oral nystatin is not absorbed, hence it is unlikely that systemic adverse reactions would be expected from this drug. The few adverse effects that were previously reported among children and adults, such as vomiting, diarrhea, and allergic reaction [5, 24, 54] were not found in our study.

### Study limitations and further suggestions

To date, this study is the first randomized controlled trial in our nation assessing the effectiveness of nystatin prophylaxis in preterm VLBW infants. In our study, no newborn infants under 28 weeks’ gestational age were recruited due to low survival related to limited resources in our NICU during the study period. Further study is required to assess the effect of nystatin prophylaxis in this particular gestational age group. It is notable that this study was conducted 8 years ago. Since the time of recruitment for this trial, our NICU has implemented a number of changes including improvements in management of respiratory care, hemodynamic instability and a comprehensive infection control program including rationalization of use of antibiotics. We acknowledge that these changes may have significant impact on the current validity of the results of this trial in the context of current practices within our NICU.

In the setting of a unit with high SFI incidence, this study has shown that nystatin prophylaxis is effective in preventing colonization and can potentially decrease the risk of SFI. Since some neonatal centers in Indonesia still face financial limitations and difficulties in maintaining intravenous access for systemic antifungal prophylaxis, the outcomes of this study have potential significance in enhancing clinicians understanding about the possible benefits of using nystatin as an alternative agent for fungal prophylaxis in a neonatal intensive care setting in Indonesia.

## Conclusion

Nystatin appears to be an effective and safe alternative prophylactic antifungal medication that reduces fungal colonization. Although reduced infection rates did not reach statistical significance, nystatin prophylaxis demonstrated a potential protective effect against SFI among VLBW preterm infants. Further studies are still required to assess the efficacy of nystatin prophylaxis among newborns under 28 weeks’ gestational age.

## Data Availability

All data generated or analyzed during the current study are included in this article.

## Abbreviations

CSF: Cerebrospinal fluid
ELBW: Extremely low birth weight
GI: Gastrointestinal
LOS: Late onset sepsis
NAC: Non-albicans Candida
NEC: Necrotizing enterocolitis
NICU: Neonatal intensive care unit
SFI: Systemic fungal infection
VLBW: Very low birth weight
